# Characterisation of the main drivers of intra- and inter- breed variability in the plasma metabolome of dogs

**DOI:** 10.1007/s11306-016-0997-6

**Published:** 2016-03-08

**Authors:** Amanda J. Lloyd, Manfred Beckmann, Kathleen Tailliart, Wendy Y. Brown, John Draper, David Allaway

**Affiliations:** Institute of Biological Environmental and Rural Sciences, Aberystwyth University, Aberystwyth, SY23 3DA UK; School of Environmental and Rural Science, University of New England, Armidale, NSW 2351 Australia; WALTHAM Centre for Pet Nutrition, Freeby Lane, Waltham-on-the-Wolds, Melton Mowbray, Leicestershire, LE14 4RT UK

**Keywords:** Metabolomics, Plasma, Metabolite fingerprinting, Multivariate data analysis, Intra-breed variability, Inter-breed variability

## Abstract

**Introduction:**

Dog breeds are a consequence of artificial selection for specific attributes. These closed genetic populations have metabolic and physiological characteristics that may be revealed by metabolomic analysis.

**Objectives:**

To identify and characterise the drivers of metabolic differences in the fasted plasma metabolome and then determine metabolites differentiating breeds.

**Methods:**

Fasted plasma samples were collected from dogs maintained under two environmental conditions (controlled and client-owned at home). The former (n = 33) consisted of three breeds (Labrador Retriever, Cocker Spaniel and Miniature Schnauzer) fed a single diet batch, the latter (n = 96), client-owned dogs consisted of 9 breeds (Beagle, Chihuahua, Cocker Spaniel, Dachshund, Golden Retriever, Greyhound, German Shepherd, Labrador Retriever and Maltese) consuming various diets under differing feeding regimens. Triplicate samples were taken from Beagle (n = 10) and Labrador Retriever (n = 9) over 3 months. Non-targeted metabolite fingerprinting was performed using flow infusion electrospray-ionization mass spectrometry which was coupled with multivariate data analysis. Metadata factors including age, gender, sexual status, weight, diet and breed were investigated.

**Results:**

Breed differences were identified in the plasma metabolome of dogs housed in a controlled environment. Triplicate samples from two breeds identified intra-individual variability, yet breed separation was still observed. The main drivers of variance in dogs maintained in the home environment were associated with breed and gender. Furthermore, metabolite signals were identified that discriminated between Labrador Retriever and Cocker Spaniels in both environments.

**Conclusion:**

Metabolite fingerprinting of plasma samples can be used to investigate breed differences in client-owned dogs, despite added variance of diet, sexual status and environment.

**Electronic supplementary material:**

The online version of this article (doi:10.1007/s11306-016-0997-6) contains supplementary material, which is available to authorized users.

## Introduction

As a consequence of selective breeding for novel and desirable traits the domestic dog (*Canis familiaris*) exhibits characteristic diversity in morphology, physiology and behaviour (Belyaev 1979; Wayne 2001). Initially, this led to the formation of distinct functional classes such as herding, hunting and guarding. Traits were then developed through restricted breeding programmes and have since been modified through pedigree breeding standards (Sutter et al. 2004). A by-product of this closed population structure and process has been that many dog breeds suffer from a high incidence of inherited disorders (Cruz et al. 2008). It is likely that other, more subtle and unintentional consequences of selective breeding may have resulted in additional changes to metabolic regulation that may impact on the plasma metabolite fingerprint of different dog breeds.

The process of metabolite fingerprinting is based on hypothesis-free, non-targeted and unbiased measurements (Draper et al. 2013; Fuhrer and Zamboni 2015). These often provide only relative quantification and aim to detect as many components in the sample as possible. Currently, global metabolite profiling and fingerprinting are achieved using high-content analytical platforms, such as ^1^H nuclear magnetic resonance (NMR) spectroscopy and mass spectrometry (MS). The latter can be used for profiling via direct infusion (DI) or flow infusion (FI) of samples or extracts, or coupled to a chromatographic or electrophoretic separation [i.e., gas chromatography (GC)-MS, liquid chromatography (LC)-MS, or capillary electrophoresis (CE)-MS] as reviewed recently (Dunn et al. 2011; Scalbert et al. 2014). One of the strengths of this type of data-driven approach is that it enables investigations of complex, nonlinear, interactive multivariate systems which are difficult to control or where no clear hypothesis exists (Kell and Oliver 2004).

Urine may not be the most suitable sample to characterize breed differences (Beckmann et al. 2010) potentially as this fluid may be dominated by microbiota-derived metabolites (Wang et al. 2007). It is likely that blood-based metabolite fingerprinting may provide more relevant data for determining “host-derived” endogenous metabolic variance between dog breeds. Previously, the plasma metabolome has provided data relevant to specific metabolic adaptations of species and also has identified differences between individuals (Allaway et al. 2013; Colyer et al. 2011). However, these data were derived from studies undertaken under controlled environmental conditions. It is likely that to achieve accurate interpretation of metabolomics data from client-owned dogs, future study designs will need to factor the major drivers of variance in the metabolome.

The primary aim of this current study was to use a large-scale non-targeted metabolomic approach to investigate the main drivers of intra- and inter- breed variation under different conditions (controlled diet and environment versus variable diet and in-home environments).

## Materials and methods

### Animal maintenance

In the controlled environment study [WALTHAM Centre for Pet Nutrition (WCPN)] a single fasted (>12 h) blood sample was collected from individual adult Labrador Retrievers (LR, n = 12), Cocker Spaniels (CS, n = 12) and Miniature Schnauzers (n = 9). The dogs were all housed in small groups in the purpose-built, environmentally-enriched facilities at the WCPN, in accordance with the Centre’s research ethics and UK Home Office Regulations. Dogs had free access to water and were fed Chappie^®^ Original dry dog food, for at least 4 weeks before and throughout the study at energy levels to maintain adult body weight (see electronic supplementary material S1 for dog details).

The client-owned dog study involved 96 dogs from 9 breeds living at home and recruited with owner consent. Samples were taken in accordance with the research ethics policy of the University of New England (UNE), Australia. The breeds were Beagle (Be, n = 14), Chihuahua (Ch, n = 5), Cocker Spaniel (CS, n = 11), Dachshund (Da, n = 7), German Shepherd (GS, n = 12), Golden Retriever (GR, n = 10), Greyhound (Gh, n = 13), Labrador Retriever (LR, n = 14) and Maltese (Ma, n = 10) (see electronic supplementary material S2 for dog details). These dogs were maintained on different diets and fed according to their owners feeding regimen and amounts. Blood was taken on one occasion for all breeds except for LR and Be, where for some individuals three separate fasted blood samples were collected at different time-points over several months (see electronic supplementary material S3 for dog details). When comparing the 9 breeds single plasma samples from individuals were analysed and where triplicates were available, the first collected sample was chosen (see S7 for structure and workflow of the studies described here).

### Collection and analysis of metadata

Metadata were collected for the client-owned dog study (UNE) to characterize the individual dogs and aid interpretation of inter- and intra- individual differences in metabolomic profiles (electronic supplementary material S2). Seven different factors were used to classify plasma samples. Individuals were assigned to at least two groups for each factor. For some factors the groups were discrete (e.g. male and female for ‘gender’) but for others involving weights, body scores and diets, we chose ranges to define the groups, aiming to create a balanced model for statistical purposes while maintaining biological relevance of the groups. The classes were breed, age category (10–24, 25–48, 49–72 and 73+ months), gender (male and female), sexual status within gender (entire and neutered), weight category (w < 10 kg, 10 ≤ w < 20 kg, 20 ≤ w < 30 kg, w ≥ 30 kg), body condition score using a 9 point score (Laflamme 1997) (reclassified into: 2, 2.5, 3, 3.5 and 4+) and owner controlled current diet (described by the owner) which was re-classified into nine diet groups (see electronic supplementary material S2 for diet details).

The impact of variance associated with each of the seven factors was investigated individually by Principal Component-Linear Discriminant Analysis (PC-LDA) on metabolite fingerprint data using a different meta-data factor for class labelling in each model (electronic supplementary material S4). Additionally, Pearson’s *χ*^2^ test was performed on the dog metadata (prior to fingerprinting) to determine whether any of these meta-data factors were associated which may influence any discrimination seen by PC-LDA. Each categorical trait was randomly assigned a numeric value and for each possible binary comparison (n = 21) a contingency table was populated from the categorical numeric vectors, and Pearson’s *χ*^2^ test for count data performed. Values displayed were the un-corrected *p* value computed from the asymptotic *χ*^2^ distribution of the test statistic. *p* values which were significant after Bonferroni adjustment, to maintain an overall 5 % error rate (*p* < 0.0024) were indicated. The base function chisq.test (R Version 3.1.2) was used for performing the *χ*^2^ test.

### Blood collection and plasma preparation

Blood was collected for both RNA and metabolite fingerprinting analysis. In keeping with reduction and refinement principles, the sample volume required was minimised by filtering blood to collect leukocytes for RNA extraction prior to centrifugation of the leukocyte-depleted blood filtrate to obtain plasma. Fasted (>12 h) blood samples (up to 9 ml), collected from the jugular vein in EDTA tubes (BD Diagnostics Vacutainer, 10 ml 367,525), were mixed by inversion and passed through a LeukoLOCK™ filter [Ambion^®^ LeukoLock™ Fractionation & stabilisation Kit (No. 1933)] using a 25G needle into an evacuated plain tube (BD Diagnostics Vacutainer, 10 ml 366636) on ice. The leukocyte-depleted blood sample was kept on ice until centrifuged (2000×*g* for 15 min). Plasma samples were collected and stored, either on dry ice for transport or for long-term storage at −80 °C until analysis.

Plasma was extracted for metabolomic analysis using the following method. Aliquots of thawed leukocyte-depleted plasma samples (200 μl) were added to 1520 µl of pre-chilled, de-gassed methanol/chloroform [4/1] in 2 mL Eppendorf tubes containing a microspoon of glass beads, vortexed, shaken for 20 min at 4 °C and then centrifuged for 5 min at 22,000×*g* at 4 °C. Extracted plasma samples were stored at −80 °C until analysis.

### Flow infusion electrospray-ionization mass spectrometry (FIE-MS)

Aliquots of extracted plasma (100 μl) were transferred into 2 mL Eppendorf tubes and dried under vacuum in a Speed-Vac vacuum concentrator (UNIVAPO 150H with a UNIJET II refrigerated aspirator). Samples were reconstituted in methanol/water [7/3], vortexed, shaken for 20 min at 4 °C and centrifuged for 5 min at 22,000×*g* at 4 °C. Supernatant (50 µl) was transferred into a HPLC vial containing a 0.2 ml flat bottom micro insert (Chromacol). Samples were randomised to avoid bias to the position of the samples within injection order especially for the triplicated samples. All samples were run together immediately after machine calibration but split into batches over consecutive days if necessary, to minimise the need for complex pre-processing steps and quality controls. FIE-MS was carried out as described previously (Beckmann et al. 2008; Fave et al. 2011). Data were acquired in alternating positive and negative ionization modes and over four scan ranges (15–110 mass-to-charge ratio (*m/z*); 100–220; 210–510; 500–1200 *m/z*), with an acquisition time of 5 min, on a LTQ linear ion trap (Thermo Electron Corporation, San Jose, CA, US). The resulting mass spectrum was the mean of 20 scans about the apex of the infusion profile. Prior to analysis, raw data signal acquisition and data pre-processing was performed in four steps. Raw data dimensionality was reduced by electronically extracting signals with ±0.1 Da mass accuracy, minimizing mass accuracy effects. Background subtraction of individual sample-attributed ion intensity as a simple baseline correction. These steps were followed by log_10_ transformation to reduce data-set variance and then normalization to total ion current (TIC) providing relative ratios of *m/z* signal abundance.

### Data analysis, sample classification and selection of potentially explanatory signals

Data mining was carried out by following the FIEmspro workflow validated previously (Enot et al. 2008) (URL http://users.aber.ac.uk/jhd/). Principal component analysis (PCA) was used to reduce data dimensionality and was followed by PC-LDA. Plots of the first two discriminant functions (DFs) allowed visualization of class separation. Random forest (RF) was employed in the analysis of the multivariate data and the RF classification ‘margin’, along with the area under the receiver operating characteristic (ROC) curve (AUC) and accuracy (ACC) were used to assess classification performance (Enot et al. 2008). Models were deemed adequate overall if RF margins >0.2 and AUC and/or ACC values >0.8, thresholds which we have implemented in previous publications (Enot et al. 2008).

A combination of RF, AUC and student’s *t* test were used to highlight potentially explanatory signals responsible for discriminating between sample classes in a full feature rank list (Enot et al. 2008). RF feature selection was performed by calculating Importance Scores, being the mean decrease in accuracy over all classes when a feature is omitted from the data. AUC used the area under curve of the sensitivity (true-positive rate) against the specificity (false-positive rate) and student’s *t*-test ranked the features by the *p* values.

Randomized re-sampling strategies using bootstrapping were applied in the process of classification and feature selection to counteract the effect of any unknown, structured variance in the data. We used 100 bootstraps in pair-wise comparisons for each of the applied statistical operations with 2/3 of data as training and 1/3 as test set. RF was set to ntree = 1000 for each bootstrap which is adequate considering the dimensionality of data.

Pearson correlation coefficients between selected variables were calculated using the function *cor* >|0.7| were considered to belong to a cluster indicative of different ionization or potential breakdown products of a single metabolite.

### Targeted accurate mass analysis and annotation of FIE-MS signals

Selected nominal mass bins were investigated further using targeted Nano-Flow (TriVersaNanoMate, AdvionBioSciences Ltd, UK) LTQ-Fourier Transform-Ion Cyclotron Resonance Ultra-Mass-Spectrometry (FT-ICR-MS; where Ultra refers to the high-sensitivity ICR-cell) as reported previously (Lloyd et al. 2011a, b). Samples were prepared as for FIE-MS and three additional plasma pools from randomized groups of dogs were prepared and reconstituted in methanol/water (80/20, v/v).

For metabolite signal identification, the accurate mass values were then queried using MZedDB, an interactive accurate mass annotation tool which can be used directly to annotate signals by means of neutral loss and/or adduct formation rules (Draper et al. 2009). FIE-MS^*n*^ was employed for further metabolite signal identification with the scan window set for 20 scans, an isolation width of 1 *m/z* and using normalized collision energy of 30 V. An activation coefficient ‘Q’ of 0.250 was chosen and an activation time of 30 ms, with wideband activation turned on and a source fragmentation of 20–30 V. Mass range settings were dependent upon the molecular weight of the target ion. Metabolites were putatively annotated to MSI level 2 without chemical reference standards due to the lack of standard availability, based upon physicochemical properties and/or spectral similarity with public/commercial spectral libraries (Lipid Maps, HMDB, Metlin and Massbank (Horai et al. 2010; Sana et al. 2008; Sud et al. 2007; Wishart et al. 2009).

## Results and discussion

### Canine breeds can be differentiated by the plasma metabolome when housed under controlled environmental conditions

**Fig. 1:**
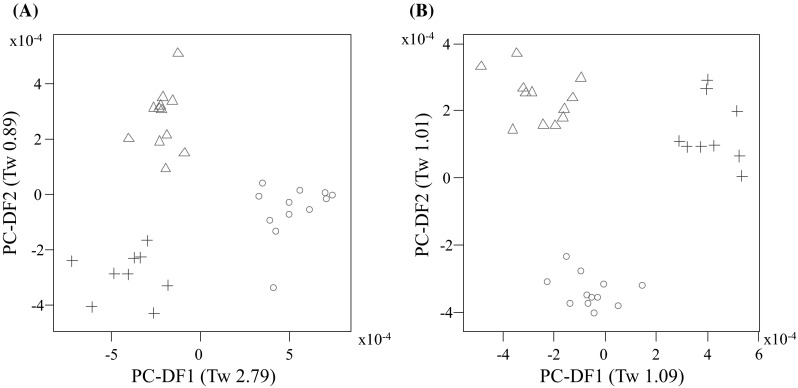
Principal Component-Linear Discriminant Analysis of Flow Infusion Electrospray-ionization Mass Spectrometry (FIE-MS) fingerprints (15–1200 *m/z*) of Labrador Retriever (LR), cocker spaniel (CS) and Miniature Schnauzer plasma samples from the WCPN study **a** positive ionization mode; **b** Negative ionization mode (1264 features). Where *circle* CS; *triangle* LR; *plus* Miniature Schnauzer. Eigenvalues (Tw values) are given in brackets

Fasted plasma samples from Labrador Retriever (n = 12), Cocker Spaniel (n = 12) and Miniature Schnauzers (n = 9) in the controlled environment study (WCPN: electronic supplementary material S1 for dog details) were analysed by non-targeted metabolite fingerprinting (FIE-MS) to generate nominal mass data in positive and negative ionization modes from *m/z* 15–1200. FIE-MS fingerprints were subjected to PCA followed by PC-LDA (Fig. 1). Discrimination was considered adequate for Eigenvalues (Tw) of >2.0 and poor for Tw <1.0 (Enot et al. 2008). In positive mode, PC-DF1 (Tw 2.79) indicated that the main source of variance was between Cocker Spaniel and the other two breeds, (Fig. 1a). Separation of plasma from Labrador Retrievers and Miniature Schnauzers was evident in PC-DF2 (Fig. 1a), however the Tw was <1.0. In negative mode (Fig. 1b), the main source of variance was between Miniature Schnauzers and the two other breeds, however the discrimination was not considered adequate. Overall, the positive mode data appeared to discriminate between the breeds more so than the negative mode data.

Previously, urinary metabolomics identified that healthy LR and Miniature Schnauzer dogs in the same environment and fed the same diets can be discriminated on a breed basis (Beckmann et al. 2010; Viant et al. 2007). In the present study, LR and Miniature Schnauzer dogs in the same environment and fed the same diets could not be discriminated using fasted plasma metabolome data (Fig. 1). As the plasma metabolome may better reflect aspects of endogenous metabolism, in this pairwise comparison these observations may be used to suggest that urine metabolome data, dominated by gut microbiota, are more informative of breed-associated microbiota than of breed genetics (Wang et al. 2007). However, breed effects were seen, with CS being strongly discriminated from LR and Miniature Schnauzer when modelling positive ionization mode plasma data. This might not have been anticipated if size was a dominant feature as CS represented a medium-sized dog, whilst LR represented larger, and Miniature Schnauzers, smaller breed sizes. This may indicate that, in this comparison, a breed effect may have been dominant to a size effect.

### The plasma metabolome of client-owned dogs can differentiate between breeds despite intra-individual variation reflecting environmental variability

As daily food intake results in acute changes to both urine and blood metabolome in humans (Jin et al. 2011; Lloyd et al. 2011a, b; Martinez-Lopez et al. 2014; Urpi-Sarda et al. 2009), it was thought valuable to determine whether uncontrolled dietary exposure would confound metabolome modelling of breed differences. To determine whether individuals maintained a relatively stable fasted plasma metabolome or whether the metabolome was highly variable and prone to environmental noise, multiple plasma samples were taken from dogs over a period of several months. To determine whether the plasma chemical fingerprint of an individual dog within its normal home environment was representative of that dog, triplicate samples of nine Labrador Retriever (LR) and 10 Beagle (Be) dogs, collected over a period of 3 months, were analysed for inter- and intra- individual differences (see electronic supplementary material S2 and S3 for dog details).

**Fig. 2:**
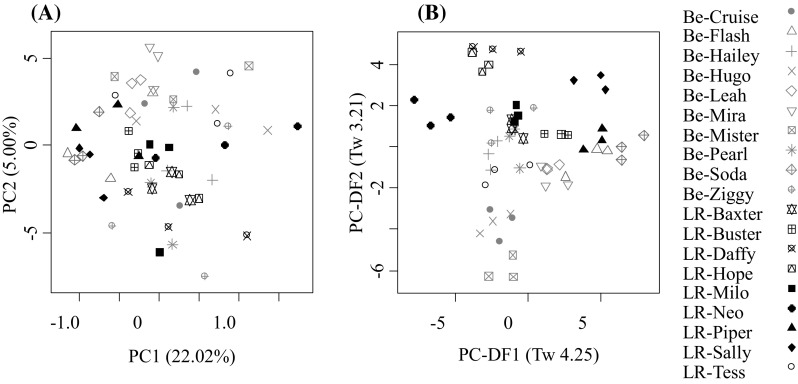
Principal component (PC) analysis followed by PC-linear discriminant analysis of flow infusion electrospray-ionization mass spectrometry (FIE-MS) fingerprints (15–1200 *m/z*) of Beagle (Be) and Labrador Retriever (LR) from the UNE study **a** PCA, positive ionization mode; **b** PC-LDA, positive ionization mode. Where *Pink* denotes Be and *black* denotes LR and *symbols* represent triplicate samples from the same dog. Eigenvalues (Tw values) are given in brackets (Color figure online)

Positive mode FIE-MS fingerprints were created and subjected to PCA (Fig. 2a) followed by PC-LDA with individual as the class structure (Fig. 2b). The majority of the individual LR triplicate samples clustered at the level of the individual in both PCA and PC-LDA. In comparison, the scores plots of the Be triplicate samples showed increased levels of intra-individual variation. Despite this intra-individual variation, a trend of breed separation was observed in both the unsupervised PCA and PC-LDA when using individual rather than breed as a class structure. Further, when the FIE-MS fingerprints were subjected to PCA for each breed separately the  % variance explained by the first two PCs did not differ greatly between the LR and Be (data not shown), suggesting similar amount of intra-breed variance. This suggests that breed may be a major driver of metabolome variance in client-owned dogs and that single blood samples from individual dogs may be sufficient to support studies characterising breed.

One possibility to explain similar intra-breed variance, when intra-individual variance was greater in Be is that although LR dogs consumed different diets, they did so consistently through the week and samples just happened to be collected on the same weekday. In comparison, the majority of the Be dogs followed inconsistent daily diets: 6 of the 10 individuals consumed a dry diet 4–5 nights a week, and consumed different ‘meaty’ meals on the other nights; the diet of 2 other Be dogs changed occasionally over the week. This variability was exacerbated by sampling individual dogs on different weekdays. When FIE-MS fingerprints of the Be dogs collected on different days were subjected to PCA it was noted that the  % variance explained by the first two PCs increased dramatically for the samples collected later in the week (data not shown). Thus both inconsistent dietary patterns of the Be breed and inconsistent sampling days may partly contribute to the increased intra-individual variability compared with the LR dogs. Despite this source of metabolome variability, clear breed separation was still evident (Fig. 2b), suggesting that the sampling method was adequate to determine whether breed is a major driver of metabolome variance in client-owned dogs.

### Breed and gender-associated differences are the main causes of variance in client-owned dogs

Having shown natural separation between Be and LR breeds using randomly sampled fasting plasma obtained from dogs maintained in an uncontrolled environment we then sought to identify the main drivers of metabolome variance in a wider range of breeds using client-owned dogs. In addition to Be and LR, we introduced Ch, CS, Da, GR, Gh, GS and Ma making the cohort up to 96 dogs (electronic supplementary material S2 for dog details). Seven different factors (age, body condition score, breed, diet, gender, within gender sexual status and weight) were used to classify plasma samples (electronic supplementary material S4). Eigenvalues (Tw) were used as a classification metric, with breed providing the largest discriminating factor (Tw values >2) in data from both ionisation modes. Weight and diet in both ionisation modes, together with within gender sexual status in negative ionisation mode were the only other classifiers resulting in near-to-adequate models (Tw values 1.4–1.7). Pearson’s *χ*^2^ test analysis was performed to determine whether any of these meta-data factors (prior to fingerprinting) were associated (electronic supplementary material S5), which may have influenced the discrimination evident in electronic supplementary material S4. As expected, gender and within gender sexual status were highly associated. A gender effect has been previously observed in adult neutered dogs, as a secondary driver of variance to breed in controlled environmental conditions (Beckmann et al. 2010). As the numbers of entire and neutered dogs were not balanced within or between breed, further analysis into the effects of gender and within gender sexual status were not investigated.

Breed, weight and diet were also reasonably associated (*p* < 0.0024), as expected considering the limited size range within breed. From the breeds chosen in this study, it is not possible to interrogate the association between genetic similarity and size similarity, and it is not possible to say whether genetics or physiology was the major influence on the plasma metabolome.

The study design was amenable to investigations of the effect of breed and diet and these were analysed further. PC-LDA plots of plasma samples represented by FIE-MS data using breed and diet class structures are shown in electronic supplementary material S6. It was found that individuals clustered by breed, which supports the view that breed is an important driver of variance in the plasma metabolome of client-owned dogs. However, only 3 of the 9 breeds (Ch, Ma and Gh) were consistently discriminated from the majority of other breeds in the study (electronic supplementary material S6 A and B). Two of the ‘small breed dogs’ Ch and Da were similar, in that they both clustered away from the other breeds on DF1 in both positive and negative ionisation mode (electronic supplementary material S6 A and B). Gh also separated from the other dog breeds, on DF2 in positive mode and DF1 in negative mode. Random Forest (RF) margins, AUC values and ACC modelling scores for all breeds were calculated, in pair-wise comparisons (Table 1). In accordance with the PC-LDA, only Ch, Da and Gh were discriminated from the majority of other breeds. Ch appeared most different to the other breeds: in comparisons with 7 of the 8 other breeds the RF margin value was >0.2 in positive and negative mode data and appeared most similar to Da. The plasma metabolome of the Da and Gh breeds was able to be discriminated from 6 of the other 8 breeds, with RF margin values >0.2 in at least one ionisation mode (Table 1). The plasma metabolome of the remaining breeds could not be adequately discriminated from at least five other breeds (RF margins <0.2, data not shown). Previous data from urinary metabolome analysis in client-owned male dogs distinguished the beagle as a distinct breed from those others tested (Beckmann et al. 2010). Here, the beagle was not readily discriminated from the majority of other breeds using mass spectral fingerprinting of plasma. Assuming that the urinary metabolome is dominated by metabolic activity of the gut microbiota and the plasma metabolome is dominated by host physiology, this observation may be interpreted to suggest that beagles have a unique gut microbiota, contributing to the differences observed previously. However, other factors exist, including that the breeds used in the comparison were not the same and that the genetic heritage of US and Australian beagles may be different.

**Table 1:** Three ‘robustness’ output statistics Random Forest (RF) classification ‘margin’, area under the receiver operating characteristic (ROC) curve (AUC) and accuracy (ACC) of flow infusion electrospray-ionization mass spectrometry (FIE-MS) fingerprints (15–1200 *m/z*) using ‘breed’ and ‘diet’ as the class structure

Pair-wise comparison	Positive			Negative		
	ACC	AUC	RF margin	ACC	AUC	RF margin
Ch vs. Be	**0.89**	**0.98**	**0.49**	**0.9**	**1**	**0.51**
Ch vs. CS	0.79	**0.99**	**0.34**	**0.85**	**0.98**	**0.35**
Ch vs. Da	0.69	**0.9**	0.13	0.76	**0.93**	0.18
Ch vs. Gh	0.79	**0.97**	**0.37**	**0.86**	**0.98**	**0.42**
Ch vs. GR	**0.88**	**0.98**	**0.33**	**0.9**	**1**	**0.39**
Ch vs. GS	**0.9**	**1**	**0.46**	**0.93**	**1 0**	**47**
Ch vs. LR	**0.9**	**1**	**0.5**	**0.92**	**0.99**	**0.48**
Ch vs. Ma	0.71	**0.94**	**0.23**	**0.85**	**0.99**	**0.31**
Da vs. Be	0.73	**0.88**	**0.26**	**0.8**	**0.95**	**0.29**
Da vs. Ch	0.69	**0.9**	0.13	0.76	**0.93**	0.18
Da vs. CS	0.72	**0.87**	0.17	0.75	**0.93**	**0.2**
Da vs. Gh	0.77	**0.95**	**0.23**	0.72	**0.92**	**0.21**
Da vs. GR	0.76	**0.92**	0.18	**0.93**	**0.99**	**0.32**
Da vs. GS	0.68	**0.89**	0.18	**0.82**	**0.95**	**0.25**
Da vs. LR	0.72	**0.85**	**0.22**	0.71	**0.88**	**0.22**
Da vs. Ma	0.71	**0.87**	0.13	0.77	**0.94**	0.16
Gh vs. Be	**0.9**	**0.97**	**0.26**	**0.83**	**0.94**	**0.21**
Gh vs. Ch	0.79	**0.97**	**0.37**	**0.86**	**0.98**	**0.42**
Gh vs. CS	**0.85**	**0.97**	0.19	**0.85**	**0.95**	**0.2**
Gh vs. Da	0.77	**0.95**	**0.23**	0.72	**0.92**	**0.21**
Gh vs. GR	**0.88**	**0.96**	**0.25**	**0.93**	**0.99**	**0.36**
Gh vs. GS	0.75	**0.88**	0.13	**0.83**	**0.92**	0.15
Gh vs. LR	0.73	**0.84**	0.12	**0.8**	**0.91**	0.16
Gh vs. Ma	**0.87**	**0.98**	**0.22**	**0.86**	**0.98**	**0.23**
4 vs. 1	0.72	**0.81**	**0.28**	0.74	**0.94**	**0.33**
4 vs. 2	0.67	0.75	0.1	0.74	**0.83**	0.15
4 vs. 3	0.67	0.73	0.08	0.7	0.77	0.1
4 vs. 5	0.72	**0.83**	0.1	0.75	**0.86**	0.14
4 vs. 6	0.67	0.58	0.17	0.68	0.73	0.18
4 vs. 7	0.72	0.72	**0.22**	0.72	0.73	**0.22**
4 vs. 8	0.71	0.42	0.14	0.69	0.64	0.18
4 vs. 9	0.67	0.72	0.18	0.7	**0.87**	**0.22**

Highlighted in bold are RF margin values >0.2; AUC >0.8; ACC >0.8

Breeds: *Be* Beagle, *Ch* Chihuahua, *CS* Cocker Spaniel, *Da* Dachshund, *GR* Golden Retriever, *Gh* Greyhound, *GS* German Shepherd, *LR* Labrador Retriever, *Ma* Maltese. Diets: 1, Dry; 2, Mince and dry; 3, Mince, dry and bones/meat; 4, Mince, dry and scraps; 5, Mince, dry, meat/bones and scraps; 6, Mince, dry and meat; 7, Dry, bones and meat; 8, Dry, bones, meat and scraps; 9, Meat and bones

When ‘diet’ was used as the class structure in a PC-LDA (electronic supplementary material S6 C and D) only diet 5 (Mince, dry food, meat/bones and scraps) and diet 4 (Mince, dry food and scraps) had a discernible impact on the plasma metabolome (clustering on opposite sides of the PC-LDA plot: S6C and D). However, these data need to be interpreted with care in relation to diet 5 as 11 of the 12 dogs following this diet were of a single breed (Gh), which accounted for the significant *p*-value in electronic supplementary material S5. In contrast, a number of breeds were exposed to diet 4 which reflects the large degree of metabolome variance associated with this factor. RF margins, AUC values and ACC modelling scores for the pair-wise comparisons of diet 4 with the other 8 diets are shown in Table 1. The plasma metabolome of dogs following diet 4 (Mince, dry food and scraps) appeared statistically different to diet 1 (Dry food) and diet 9 (Meat and bones), thus it could be suggested that the metabolome differences seen here could be attributed to the intake of table scraps. Regarding the impact of diet on the fasted plasma metabolome the data indicate that variance due to the diet (albeit in a subjective and complex class structure) explained less of the variance than breed in both positive and negative ion spectra. We conclude that whilst diet was a confounding factor for both intra-individual reproducibility and inter-breed comparisons, it was not dominant to the breed effect in this study. Other factors that may need to be considered as important to control in future studies include gender, within gender sexual status and weight, whilst age and body condition score were not considered significant drivers of variance within this cohort.

### Environmentally robust plasma metabolome differences exist between dog breeds

We next sought to investigate whether breed information is sufficiently dominant to be consistent between dogs fed the same diet in a controlled environment and client-owned dogs, with the added variance of diet, sexual status and environment. Two breeds, LR and CS were common to both the WCPN (controlled) and UNE (client-owned) studies.

**Fig. 3:**
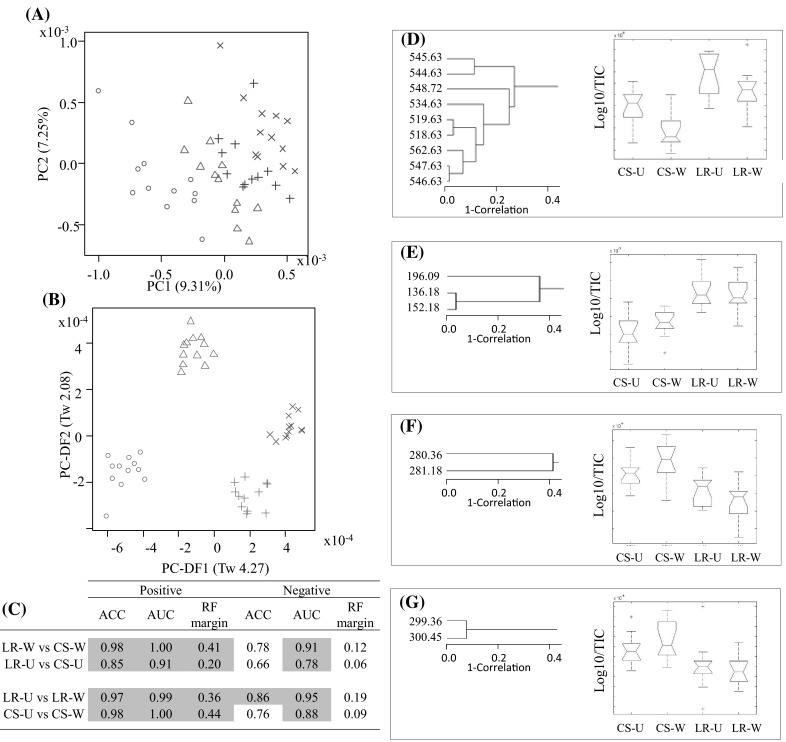
Flow Infusion Electrospray-ionization Mass Spectrometry (FIE-MS) fingerprints (15–1200 *m/z*) of fasting plasma samples from Labrador (LR) and Cocker Spaniel CS from the WCPN (W) and UNE (U) study **a** Principal Component (PC) Analysis, positive ionization mode where *circle* CSW; *triangle* LRW; *plus* CSU; *crossmark* LRU. Eigenvalues (Tw values) are given in brackets; **b** PC-Linear Discriminant Analysis, positive ionization mode. Eigenvalues (Tw values) are given in brackets; **c** Random Forest (RF) classification ‘margin’, area under the ROC (receiver operating characteristic) curve (AUC) and accuracy (ACC). Highlighted in *grey* are RF margin values >0.2; AUC >0.8; ACC >0.8; Hierarchical cluster analyses (HCA) based on the correlation coefficient (Pearson correlation method) and box-plots of the top discriminatory features in the WPCN study and UNE study **d** HCA of a cluster 1 with a box plot of *m/z* 518.63; **e** HCA of a cluster 2 with a box plot of *m/z* 136.18; **f** HCA of a cluster 3 with a box plot of *m/z* 280.36; **g** HCA of a cluster 4 with a box plot of *m/z* 299.36. TIC, Total ion count

For this comparison, data from FIE-MS fingerprints were subjected to PCA followed by PC-LDA. The data indicated that the differences between the samples derived from the two sites (UNE vs WCPN) were the main source of variance (Fig. 3a–c). The discrimination between LR and CS was evident in the second dimension of the PC-LDA (Fig. 3b) and despite the large environmental effect, breed differences were still adequate (Tw > 2.0) in this dimension.

Three ‘robustness’ output statistics (ACC, AUC values and RF margins) of metabolite fingerprinting data derived from analysis of plasma from the two WCPN dog breeds are shown in Fig. 3c. High values indicate excellent discrimination between LR and CS in positive mode FIE-MS data for both the WCPN and UNE study (Fig. 3c), despite a large environmental effect between the two studies and a large degree of environmental variance within the UNE study. Identification of metabolites that discriminated between breeds under such different environmental conditions was considered to indicate breed-associated functional differences. The top ranked signals discriminating between CS and LR in the WCPN and UNE study (*p* value <0.05 and RF Importance Score >0.002) were identified (data not shown). Several clusters of signals appeared to strongly discriminate between the breeds in both studies (Fig. 3d–g), some of which were increased in intensity in LR plasma (Fig. 3d–e) and some which were increased in intensity in CS (Fig. 3f–g). The LR-specific cluster 2 (Fig. 3d–e) was most probably an [M + K]^1+^ adduct (*m/z* 152.18) and an [M + Na]^1+^ adduct (*m/z* 136.18) due to the difference of nominal mass *m/z* 16, plus another correlated adduct (*m/z* 196.09). The two CS specific clusters (Fig. 3f–g) consisted of parent molecules and corresponding ^13^C isotopes (nominal mass difference 1 *m/z*). The cluster with the highest number of correlated signals was cluster 1 (Fig. 3d), so we selected these signals and annotated them in greater detail.

**Fig. 4:**
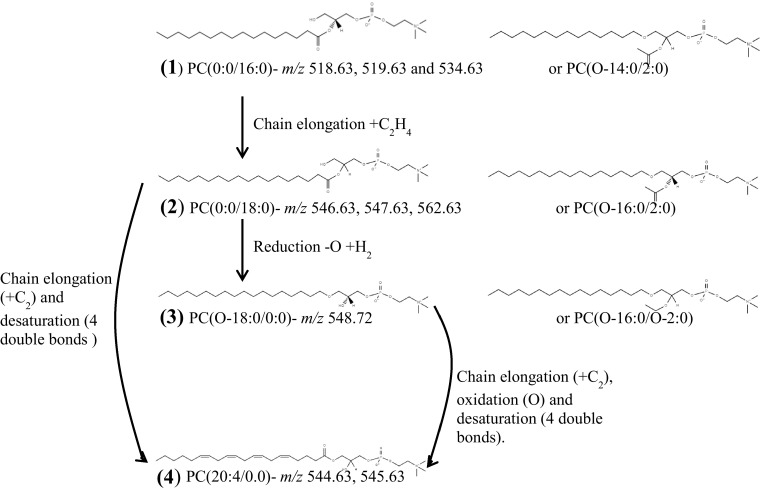
The biochemical relationships between the phosphatidylcholines (PC) in cluster 1

Using Fourier Transform-Ion Cyclotron Resonance Ultra Mass Spectrometry (FT-ICR-MS) and Flow Infusion Electrospray-Ionization Tandem Mass Spectrometry (FIE-MS^*n*^) coupled with the signal annotation tool MZedDB (Draper et al. 2009) and spectral libraries ((Lipid Maps, HMDB, Metlin and Massbank (Horai et al. 2010; Sana et al. 2008; Sud et al. 2007; Wishart et al. 2009)) the signals in cluster 1 were putatively annotated to MSI level 2 as adducts and isotopes of four structurally related phosphatidylcholines (Table 2). Phosphatidylcholines are predominantly structural lipids important in plasma membranes and lung surfactants as well as being major components in lipoproteins. Inter-breed differences in lipoproteins have been identified, with LR having particularly high levels of Low Density Lipoproteins and low levels of High Density Lipoproteins (Downs et al. 1993; Pasquini et al. 2008) compared to other dog breeds in those studies, though CS were not included. In other reports, breed differences in phosphatidylcholines in pulmonary surfactants have been identified in Be and Gh (Clercx et al. 1989). The breed difference was considered to be a likely consequence of selection for the Gh for specific athletic attributes (for example, improved ventilation to perfusion matching). It is possible to speculate that the identification of phosphatidylcholine as a robust marker of breed difference may indicate differences in lipid metabolism and lipid functionality between breeds. This finding parallels work which identified a group of lipids showing high inter-individual variance within individuals of both dogs and cats (Colyer et al. 2011). The structural similarities and possible biochemical relationships are shown in Fig. 4. A comprehensive lipidomics analysis may in the future establish whether other elements of lipid biology are different between breeds.

**Table 2:** Identification of signals explanatory of the Labrador Retriever breed by Fourier Transform-Ion Cyclotron Resonance Ultra Mass-Spectrometry (FT-ICR-MS) and Flow Infusion Electrospray-Ionization Tandem Mass Spectrometry (FIE-MS^*n*^)

Nominal mass	Accurate mass using FT-ICR-MS	Identification confirmed with FIE-MS^n^	Molecular formula and ionization product	Calculated mass	PPM Δ
518.63	518.3224	PC(0:0/16:0) or PC(O-14:0/2:0)	C_24_H_50_NO_7_P & [M + Na]^1+^ =C_24_H_50_NNaO_7_P	518.32171	1.3
519.63	519.32561	^13^C isotope of PC(0:0/16:0) or PC(O-14:0/2:0)	C_24_H_50_NO_7_P & [M + Na]^1+^ =^13^C isotope C_24_H_50_NNaO_7_P	519.32507	1.03
534.63	534.29617	PC(0:0/16:0) or PC(O-14:0/2:0)	C_24_H_50_NO_7_P & [M + K]^1+^=C_24_H_50_NKO_7_P	534.295651	0.98
546.63	546.353575	PC(0:0/18:0) or PC(O-16:0/2:0)	C_26_H_54_NO_7_P & [M + Na]^1+^ =C_26_H_54_NNaO_7_P	546.35301	1.03
547.63	547.356445	^13^C isotope of PC(0:0/18:0) or PC(O-16:0/2:0)	C_26_H_54_NO_7_P & [M + Na]^1+^ =^13^C isotope C_26_H_54_NNaO_7_P	547.35637	0.14
562.63	562.327388	PC(0:0/18:0) or PC(O-16:0/2:0)	C_26_H_54_NO_7_P & [M + K]^1+^ =C_26_H_54_KNO_7_P	562.32695	0.78
548.72	548.34718	PC(O-18:0/0:0) or PC(O-16:0/O-2:0)	C_26_H_56_NO_6_P & [M + K]^1+^ =C_26_H_56_KNO_6_P	548.34769	0.92
544.63	544.34034	PC(20:4/0:0)	C_28_H_50_NO_7_P & [M + H]^1+^ =C_28_H_51_NO_7_P	544.33979	1.06
545.63	545.34374	^13^C isotope of PC(20:4/0.0)	C_28_H_50_NO_7_P & [M + H]^1+^ =^13^C isotope C_28_H_51_NO_7_P	545.34312	1.13

Metabolites have been putatively annotated to MSI level 2 without chemical reference standards, based upon physicochemical properties and/or spectral similarity with public/commercial spectral libraries

*PC* phosphatidylcholine. *PPM∆* parts per million difference

## Concluding remarks

To summarise, a data-driven, non-targeted metabolomic analysis of plasma from various dog breeds in different locations and fed different habitual diets has enabled us to establish that factors associated with breed and gender were the main drivers of variance in fasted plasma. We use these observations to suggest that the metabolite fingerprint of an individual is primarily a function of size and genetics and that environmental factors can confound these physiological elements. Future studies may consider the implications of these findings in relation to developing an evidence base to help determine the specific nutritional requirements of different dogs.

## Electronic supplementary material

Supplementary material 1 (DOCX 11 kb)

Supplementary material 2 (XLSX 103 kb)
