# Supplementary material for: Characterisation of the main drivers of intra- and inter- breed variability in the plasma metabolome of dogs
Source: Metabolomics. 2016 Mar 8;12:72. doi: 10.1007/s11306-016-0997-6 (PMC4783439; doi:10.1007/s11306-016-0997-6)
Supplement: Supplementary file 1 — Supplementary material 1 (DOCX 11 kb) [file 11306_2016_997_MOESM1_ESM.docx]

**Electronic supplementary material**

**S**1 Dogs used in the controlled WCPN analysis

**S2** Dogs used in the uncontrolled study consisting of 9 breeds

**S3** Dogs with triplicated samples

**S4** Principal Component -Linear Discriminant Analysis (PC-LDA) Eigenvalues (Tw) where different class structures have been applied to Flow Infusion Electrospray-ionization Mass Spectrometry (FIE-MS) fingerprint data (15-1200 m/z), as indicated in the table

**S5** Pearson's Chi -squared analysis where different class structures have been applied to the dog metadata, as indicated in the table. Values are the un-corrected p-value computed from the asymptotic Chi - squared distribution of the test statistic. Cells marked with * indicate that the p-value is significant after Bonferroni adjustment, to maintain an overall 5% error rate (p < 0.0024)

**S6** Influence of diet and breed on modelling plasma samples. Principal Components-Linear Discriminant Analysis (PC-LDA) of data acquired by Flow Infusion Electrospray ionization Mass Spectrometry (FIE-MS; *m/z* 15-1200) of single replicate plasma samples collected from 96 dogs: Beagle (Be), Chihuahua **(**Ch), Cocker Spaniel (CS), Dachshund (Da), Golden Retriever (GR), Greyhound (Gh), German Shepherd (GS), Labrador Retriever (LR) and Maltese (Ma) from the UNE study. Different class structures were applied, as indicated in electronic supplementary material S2. (A) Positive mode, breed; (B) Negative mode, breed. (C) Positive mode, diet; (D) Negative mode, diet; Eigenvalues (Tw values) are given in brackets

**S7** Structure and workflow of the studies
